# CLytA-DAAO, Free and Immobilized in Magnetic Nanoparticles, Induces Cell Death in Human Cancer Cells

**DOI:** 10.3390/biom10020222

**Published:** 2020-02-03

**Authors:** María Fuentes-Baile, Daniel Bello-Gil, Elizabeth Pérez-Valenciano, Jesús M. Sanz, Pilar García-Morales, Beatriz Maestro, María P. Ventero, Cristina Alenda, Víctor M. Barberá, Miguel Saceda

**Affiliations:** 1 Hospital General Universitario de Alicante, Instituto de Investigación Sanitaria y Biomédica de Alicante (ISABIAL), C/Maestro Alonso, 10, 03005 Alicante, Spain; mariafuentesbaile@gmail.com (M.F.-B.); maripazvm@gmail.com (M.P.V.); alenda.cris@gmail.com (C.A.); 2 Unidad de Investigación. Hospital General Universitario de Elche, Fundación para el Fomento de la Investigación Sanitaria y Biomédica de la Comunidad Valenciana (FISABIO), Camí de l’Almazara, 11, 03203 Elche (Alicante), Spain; pgarcia@umh.es (P.G.-M.); barbera_vicjua@gva.es (V.M.B.); 3 Instituto de Investigación, Desarrollo e Innovación en Biotecnología Sanitaria de Elche (IDiBE), Universidad Miguel Hernández, Avda. Universidad s/n, Ed. Torregaitán, 03202 Elche (Alicante), Spain; dabe_gil@yahoo.es (D.B.-G.); elizabethpv2908@gmail.com (E.P.-V.); jmsanz@cib.csic.es (J.M.S.); bmaestro35@gmail.com (B.M.); 4 Centro de Investigaciones Biológicas Margarita Salas (Consejo Superior de Investigaciones Científicas) and Centro de Investigación Biomédica en Red de Enfermedades Respiratorias (CIBERES), C/Ramiro de Maeztu, 9, 28040 Madrid, Spain; 5 Unidad de Genética Molecular. Hospital General Universitario de Elche. Camí de l’Almazara, 11, 03203 Elche (Alicante), Spain

**Keywords:** magnetic nanoparticle, cancer therapy, reactive oxygen species, oxidative damage, cytotoxicity, choline-binding proteins

## Abstract

D-amino acid oxidase (DAAO) catalyzes the oxidation of D-amino acids generating hydrogen peroxide, a potential producer of reactive oxygen species. In this study, we used a CLytA-DAAO chimera, both free and bound to magnetic nanoparticles, against colon carcinoma, pancreatic adenocarcinoma, and glioblastoma cell lines. We found that the enzyme induces cell death in most of the cell lines tested and its efficiency increases significantly when it is immobilized in nanoparticles. We also tested this enzyme therapy in non-tumor cells, and we found that there is not cell death induction, or it is significantly lower than in tumor cells. The mechanism triggering cell death is apparently a classical apoptosis pathway in the glioblastoma cell lines, while in colon and pancreatic carcinoma cell lines, CLytA-DAAO-induced cell death is a necrosis. Our results constitute a proof of concept that an enzymatic therapy, based on magnetic nanoparticles-delivering CLytA-DAAO, could constitute a useful therapy against cancer and besides it could be used as an enhancer of other treatments such as epigenetic therapy, radiotherapy, and treatments based on DNA repair.

## 1. Introduction

Nowadays, cancer remains among the main causes of mortality [1]. Surgery followed by chemotherapy and radiotherapy are the most commonly used treatments against cancer. However, the selectivity of most drugs towards tumor cells is still too low. In addition, the low therapeutic index, the lack of treatment specificity, and the appearance of drug-resistant clonal populations of cells often reduce the effectiveness of these therapies. In addition, another problem associated with the treatment of solid tumors is the access of drugs to tumor cells which is limited by poor vascularization and necrosis [2].

Due to its accelerated metabolism, tumor cells exhibit increased free radical levels in comparison with normal cells [3]. The high levels of free radicals in tumor cells make them more vulnerable to death due to oxidative stress, so this is a putative strategy for antitumor therapies. 

A novel strategy for the treatment of solid tumors is to use enzymatic therapies based on the production of reactive oxygen species (ROS). An exogenous enzyme is directed to the tumor and then a non-toxic substrate of the enzyme is administered systemically, so that it can be converted into an active anticancer drug inside the tumors [4,5]. 

Our research group is studying the use of the D-amino acid oxidase (DAAO) from *Rhodotorula gracilis* for the treatment of cancer. DAAO catalyzes the oxidation of D-amino acids in alpha-ketoacids, ammonium, and H_2_O_2_. DAAO from yeasts present a very high catalytic activity and a stable interaction with the cofactor flavin-adenine dinucleotide (FAD) [6,7]. In addition, its substrate (D-amino acids) is not present endogenously, allowing a simple regulation of the enzymatic activity [8,9]. 

To prevent the enzyme from being degraded by the organism or not be able to reach the tumor, it is necessary to direct it specifically towards the tumor. Immobilization provides a support to the enzymes, and usually encompasses favorable conditions, such as increasing structural stability and/or enzyme specificity/activity, better kinetic properties, or extending its pH or temperature working-range [10,11]. Numerous methods have been implemented for enzyme immobilization, taking into account the intended application [12]. In this sense, magnetic nanoparticles (MNPs) have received increasing attention as enzyme carriers for biotechnological and biomedical applications [13]. 

MNPs can easily be recovered from aqueous solutions using an external magnetic field and exhibit useful properties such as high surface area-to-volume ratio, making possible an increase in the enzyme density, and the possibility to be surface modified [14]. In our study, we used a non-covalent site-specific immobilization of the enzyme, as this method uses mild conditions. To get this, we made use of the affinity tag CLytA, which is the choline-binding module of the amidase N-acetylmuramoyl-L-alanine (LytA) from *Streptococcus pneumoniae* [15]. The CLytA domain shows high affinity for choline and choline structural analogs, such as diethylaminoethanol (DEAE), and it is routinely used as an affinity tag for the single-step purification and immobilization of fusion proteins [16,17,18]. 

The chimera was specifically immobilized onto MNPs functionalized with DEAE. Both, free and immobilized, CLytA-DAAO chimera are able to induce cell death by increasing ROS production, which caused DNA damage in several colon carcinoma and pancreatic adenocarcinoma cell lines as well as in glioblastoma cell lines derived from primary cultures obtained in our laboratory directly from glioblastoma patients, at doses that are safe for non-tumor cells. Interestingly, the cell death evoked by the enzyme could be executed by apoptotic or necrotic mechanisms depending on the tumor origin. 

The reasons to select these types of tumors in our study were their frequency, mortality, and resistance to other treatments and also our laboratory experience, since we have been working in these models for many years [19,20,21,22,23]. This localized therapy reduces the dose of drug needed, improving the effectiveness and decreasing adverse effects. Finally, in this study we demonstrate that, besides its own cell-death induction capacity, this kind of therapy is also able to potentiate the effect of other treatments, such as epigenetic treatments with histone deacetylase inhibitors, radiotherapy, and Poly (ADP-ribose) polymerase (PARP) inhibitors on these poor prognosis types of cancer.

## 2. Materials and Methods

### 2.1. Cell Culture

The human pancreatic adenocarcinoma cell lines IMIM-PC-2, RWP-1, and Hs766T, the human colon carcinoma cell lines SW-480, SW-620, and HT-29, the non-tumor cell lines from human fibroblasts IMR90 and 1BR3.G, and the human ductal pancreatic cell line HPDE were donated by the Instituto Municipal de Investigaciones Médicas (IMIM, Barcelona, Spain). The glioblastoma cell lines HGUE-GB-18, HGUE-GB-37, HGUE-GB-39, and HGUE-GB-42 derived from primary cultures were established in our laboratory [24]. Differentiated mouse 3T3-L1 cells were donated by Dr. Vicente Micol of Instituto de Investigación, Desarrollo e Innovación en Biotecnología Sanitaria de Elche (IDiBE, Elche, Spain) [25]. The lymphocytes primary cultures were obtained from blood samples of non-oncological patients at “Hospital General Universitario de Elche” (HGUE). 

Colon carcinoma cell lines, pancreatic adenocarcinoma cell lines, adipocytes, and fibroblasts were maintained in Dubelcco’s Modified Eagle’s Medium (DMEM) High Glucose (Biowest, MO, USA) while glioblastoma cell lines were maintained in DMEM: Nutrient Mixture F-12 (DMEM F12) (Biowest, MO, USA). HPDE cell line was cultured in keratinocyte serum-free (KSF) medium supplemented with epidermal growth factor and bovine pituitary extract (Life Technologies, Inc., Grand Island, NY, USA) as previously described [26]. The lymphocytes primary cultures were maintained in Roswell Park Memorial Institute (RPMI) 1640 media (Biowest, MO, USA). DMEM, DMEM F-12 and RPMI 1640 media were supplemented with 10% (*v*/*v*) heat-inactivated fetal bovine serum (FBS) (Biowest, MO, USA) and 1% (*v*/*v*) of a penicillin and streptomycin mixture (Biowest, MO, USA). Cells were incubated at 37 °C in a humidified 5% CO_2_ atmosphere. 

### 2.2. Ethics Statement

Blood samples from non-oncological patients were collected from routine tests, after the signing of informed consent by the participants and with the approval of the Ethical and Clinical Research Committee of the HGUE (Elche, Spain). 

### 2.3. CLytA-DAAO Production

CLytA-DAAO was obtained and isolated as previously described [27], with minor modifications. CLytA-DAAO protein was extracted from *Escherichia coli* BL21 (DE3) [28], which was transformed with the plasmid pCPC21 [29], and purified using the QIAprep^®^ Spin Miniprep Kit (Qiagen, Hilden, Germany), following the protocol previously described [30]. This plasmid allows the overexpression of the CLytA-DAAO hybrid gene.

A single colony was grown in Luria–Bertani (LB) medium [31], supplemented with ampicillin (0.1 mg mL^−1^) in orbital agitation at 37 °C. After reaching an optical density of 0.6, the expression of CLytA-DAAO gene was induced for 14 h at 30 °C by the addition of 0.5 mM of isopropyl-β-D-1-thiogalactopyranoside (IPTG), which allows the induction of the *lac* promoter. The cells were harvested by centrifugation, resuspended in sodium phosphate buffer 20 mM, NaCl 100 mM (pH 7.0) and then, adenine-flavin di-nucleotide (FAD) was added as cofactor of DAAO protein. Cells were lysed by sonication. 

The resulting extract was centrifuged, and the supernatant obtained was purified by affinity chromatography. Purity of the eluted protein was checked by sodium dodecyl sulfate polyacrylamide gel electrophoresis [32]. 

### 2.4. CLytA-DAAO Immobilization in MNPs

DEAE-FluidMAG magnetic nanoparticles (5 mg, 200 nm diameter, Chemicell, Berlin, Germany), equilibrated in 20 mM sodium phosphate 10% glycerol (*w*/*w*), at pH 7.5, were incubated with 500 μg of purified CLytA-DAAO, under mild circular agitation for 10 min at 25 °C. Glycerol was added as described to stabilize the DAAO structure [33]. Particles were then separated with a magnet (Chemicell, Berlin, Germany), followed by repeated washing steps in the equilibrate buffer previously used. 

### 2.5. Determination of the Pure CLytA-DAAO Enzymatic Activity

D-amino acid oxidase activity of the fusion protein CLytA-DAAO was tested following the protocol described by Fonda and Anderson [34]. Formation of benzoyl-formic acid (ε_252_ = 12,253 M^−1^cm^−1^), produced by the hydrolysis of D-phenylglycine (15 mM) in 20 mM sodium phosphate buffer at pH 7.5, was determined spectrophotometrically at 252 nm, after stopping the reaction with glacial acetic acid. One unit (U) of enzymatic activity was defined as the amount of enzyme needed to produce 1 µmol of product per min at 25 °C and pH 7.5 [35]. 

### 2.6. Chemical Reagents and Treatments

The pan-caspase inhibitor carbobenzyloxy-Val-Ala-Asp-α-fluoromethylketone (Z-VAD-FMK) (Calbiochem^®^, San Diego, CA, USA) was used at 25 μM to test caspase dependent apoptosis. Chloroquine (CQ) and Spautine-1 (SP) (Sigma-Aldrich, MO, USA) were used at 10 μM to test autophagy. Necrostatine-1 (NC) (Sigma-Aldrich, MO, USA) was used at 20 μM to test necroptosis cell death. Ferrostatin-1 (Fe-1) (Sigma-Aldrich, MO, USA) were used at 10 μM to test ferroptosis cell death. Trichostatin A (TSA) (Sigma-Aldrich, MO, USA) was used at 0.1 μM and the PARP inhibitor III, 3,4-Dihydro-5[4-(1-piperindinyl)butoxy]-1(2H)-isoquinoline (DPQ) (Calbiochem^®^, San Diego, CA, USA) was used at 10 μM.

Radiotherapy treatment at 7 Gy was added to cells with a rate of 6 Gy min^−1^ using VARIANT 2100C linear accelerator in the radiotherapy Oncology Unit of the HGUE.

The Bax-inhibiting peptide, V5 (H-VPMLK-OH) (Calbiochem^®^, San Diego, CA, USA) was used at 200 μM according to the manufacturer’s instructions.

### 2.7. Cell Proliferation Assays

Cells were seeded in 96-well plates (Sarstedt, Nümbrecht, Germany) at a density of 4000 cells per well and incubated at 37 °C and 5% CO_2_ for 24 h. After this time, the different treatments were added in sextuplicate and the plate was incubated at the same conditions for 72 h. Then, 0.25 mg mL^−1^ of methylthiazolyldiphenyl-tetrazolium bromide (MTT) (Sigma-Aldrich, MO, USA) were added for 3 h. Media was removed and 200 μL of dimethyl sulfoxide (DMSO) (Sigma-Aldrich, MO, USA) was added and the plate was shaken at room temperature for 30 min to dissolve the formazan crystals [36]. Finally, the absorbance at 570 nm was measured on a Gen5™ model microplate reader (BioTeK^®^, Winooski, VT, USA). 

### 2.8. Cell Cycle Analysis

Cells were seeded in six-well plates (Sarstedt, Nümbrecht, Germany) and, after the treatment, they were harvested by trypsinization and fixed in cold ethanol (75%) for at least 30 min at −20 °C. Once the cells were fixed, they were pelleted, resuspended in 0.5 mL of Phosphate Buffered Saline (PBS) in the presence of 0.5% Triton X-100, 25 µg mL^−1^ of RNase A (Serva, Heidelberg, Germany), and 25 µg mL^−1^ of propidium iodide (Sigma Aldrich, MO, USA) and incubated for 30 min at room temperature in the dark. Finally, BD FACSCanto™ flow cytometer (Becton Dickinson & Co., Franklin Lakes, NJ, USA) was used to determine the distribution of the cells in the different phases of the cell cycle according to the DNA content. 

### 2.9. Intracellular ROS Measurement

After the cell treatment, intracellular ROS generation was determined using 2′,7′-dichlorodihydrofluorescein diacetate (H_2_DCF-DA) (Sigma-Aldrich, MO, USA), a fluorescence probe able to bind to free radicals. The test was carried out in an opaque 96-well plate (Sigma-Aldrich, MO, USA), and the probe was added at 10 µg mL^−1^ during the time that ROS production was intended to be measured. After this time, the media was removed, and the plate was washed with PBS. Finally, the fluorescence was measured in a fluorescence plate reader POLARstar Omega (BMG Labtech, Ortenberg, Germany) using excitation and emission wavelengths of 485 and 520 nm, respectively. 

### 2.10. DNA Damage Measurement

Cells were seeded in six-well plates (Sarstedt, Nümbrecht, Germany) and, after 24 h of incubation at 37 °C and 5% CO_2_, the corresponding treatments were added. At the end of the treatment time, the phosphorylated form of the histone H2A.X was determined using the “Activation Dual Detection Kit” (Merck Millipore, Darmstadt, Germany), according to the manufacturer’s instructions, and the Muse^®^ Cell Analyzer (Millipore Corporation, Darmstadt, Germany). 

### 2.11. Viability Assay

Cells were seeded in six-well plates (Sarstedt, Nümbrecht, Germany) and, after 24 h of incubation at 37 °C and 5% CO_2_, the corresponding treatments were added. At the end of the treatment time, the viability was determined using the “Count and Viability Kit” (Merck Millipore, Darmstadt, Germany), according to the manufacturer’s instructions, and the Muse^®^ Cell Analyzer (Millipore Corporation, Darmstadt, Germany).

### 2.12. Annexin V-PE Assay

Cells were seeded in six-well plates (Sarstedt, Nümbrecht, Germany) and, after 24 h of incubation at 37 °C and 5% CO_2_, cells were treated 24 h with CLytA-DAAO and D-Alanine. At the end of the treatment, the apoptotic cell death was determined using the “Annexin V and Dead Cell Kit” (Merck Millipore, Darmstadt, Germany), according to the manufacturer’s instructions, and the Muse^®^ Cell Analyzer (Millipore Corporation, Darmstadt, Germany).

### 2.13. Lactate Dehydrogenase Assay Kit

Cells were seeded in 96-well plates (Sarstedt, Nümbrecht, Germany) and, after 24 h of incubation at 37 °C and 5% CO_2_, the corresponding treatments were added. At the end of the treatment, the extracellular lactate dehydrogenase (LDH) activity was determined using the “Cytotoxicity Detection KitPLUS (LDH)” (Roche Diagnostics GmbH, Mannheim, Germany), according to the manufacturer’s instructions, and the absorbance at 492 nm was measured on a Gen5™ model microplate reader (BioTeK^®^, Winooski, VT, USA).

### 2.14. Plasma Membrane Permeability

Cells were seeded in six-well plates (Sarstedt, Nümbrecht, Germany) and, after 24 h of incubation at 37 °C and 5% CO_2_, the treatment was added. After 6 h of treatment with CLytA-DAAO, the media was replaced by PBS with 15 µg mL^−1^ of propidium iodide. The plate was kept in the dark for 30 min, and the labeled cells were photographed with a fluorescence microscope (Nikon Eclipse TE2000-U) equipped with a digital camera (Nikon DS-1QM). 

### 2.15. Statistical Analysis

The results are shown as the mean ± standard error of the mean (SEM) of at least three independent experiments. A descriptive statistic was performed with the GraphPad Prism version 7 (GraphPad Software Inc., San Diego CA, USA) calculating the mean, standard error of the mean, and standard deviation for the values. Shapiro Wilk statistical test was used to evaluate the normal distribution of the data, and to analyze the association between variables the Student’s *t* or Mann–Whitney U test were used. *p*-values lower than 0.05 were considered statistically significant. 

## 3. Results

### 3.1. CLytA-DAAO Effect in Cell Lines of Different Origin

**Figure 1:**
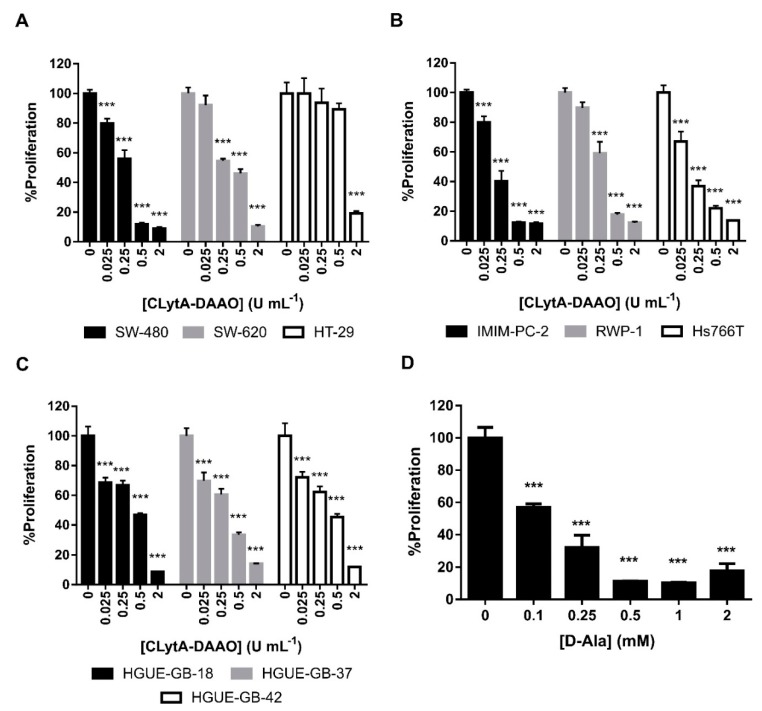
CLytA-DAAO effects on cell proliferation. (**A**) SW-480, SW-620, and HT-29 colon carcinoma cell lines, (**B**) IMIM-PC-2, RWP-1, and Hs766T pancreatic adenocarcinoma cell lines, and (**C**) HGUE-GB-18, HGUE-GB-37, and HGUE-GB-42 glioblastoma cell lines were treated with 0.025-2 U mL^−1^ CLytA-DAAO and 1 mM D-Ala for 72 h and cell proliferation was evaluated by methylthiazolyldiphenyl-tetrazolium bromide (MTT) assay. (**D**) SW-480 colon carcinoma cell line was treated with 2 U mL^−1^ CLytA-DAAO and 0.1–2 mM D-Ala for 72 h and cell proliferation was evaluated by MTT assay. Data represent the mean ± SEM of the percentages of cells with respect to control, with n≥6. *** *p* < 0.001.

To determine the IC50 of CLytA-DAAO in colon carcinoma, pancreatic adenocarcinoma and glioblastoma cell lines derived from primary cultures established in our laboratory, different concentrations of CLytA-DAAO and 1 mM D-Alanine were tested in cell proliferation MTT assays. IC50 values are shown in Table 1. Results in Figure 1A–C show that 2 U mL^−1^ of CLytA-DAAO are enough to achieve the maximum CLytA-DAAO effect in all cell lines tested. We determined, as well, the optimal concentration of D-Alanine required to trigger the anti-proliferative effects of CLytA-DAAO in these cell lines. Figure 1D shows a representative result in SW-480 cell line, and we found that D-Alanine 1 mM is enough to reach the maximal effect of the enzyme.

**Figure 2:**
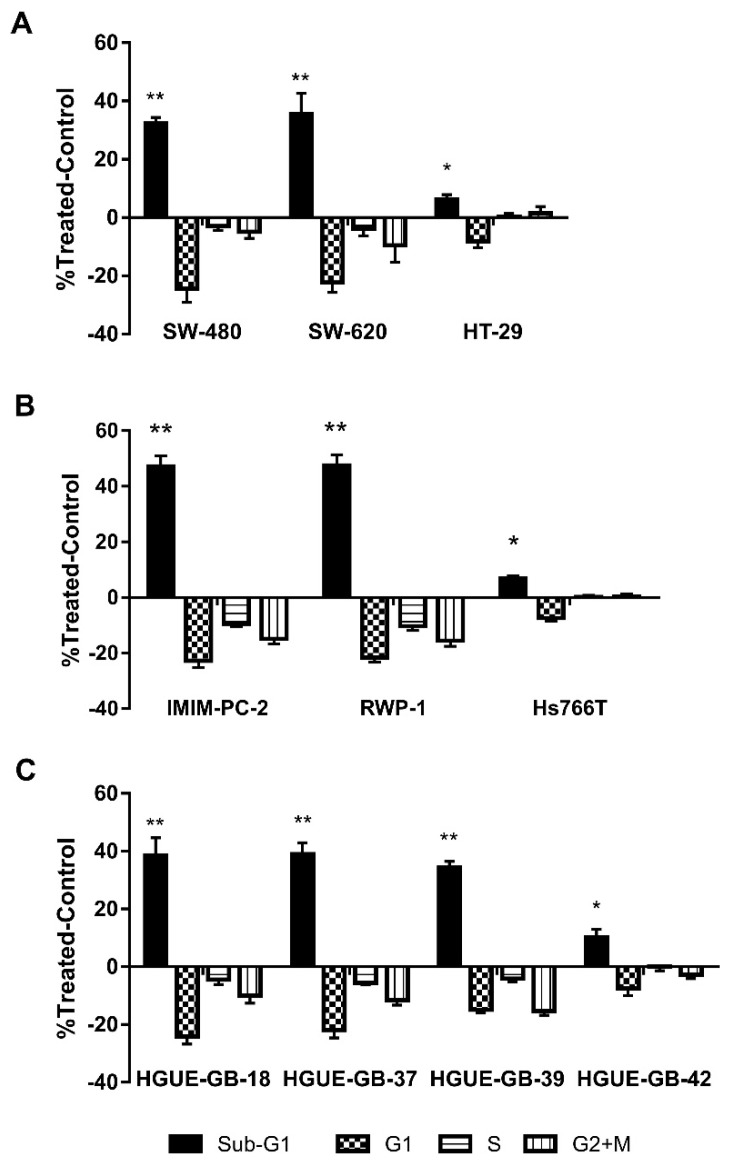
CLytA-DAAO effects on cell cycle. (**A**) SW-480, SW-620, and HT-29 colon carcinoma cell lines, (**B**) IMIM-PC-2, RWP-1, and Hs766T pancreatic adenocarcinoma cell lines, and (**C**) HGUE-GB-18, HGUE-GB-37, HGUE-GB-39, and HGUE-GB-42 glioblastoma cell lines were treated with 2 U mL^−1^ CLytA-DAAO and 1 mM D-Ala for 24 h and cell cycle distribution was determined by flow cytometry. Data represent the mean ± SEM of changes in the percentage of cells in each phase of the cell cycle compared with the untreated control, with *n* ≥ 6. * indicates *p* < 0.05 and ** *p* < 0.01.

In order to determine whether the effect of the CLytA-DAAO at 2 U mL^−1^, with 1 mM D-Alanine, was cytotoxic or cytostatic we performed a cell cycle analysis after 24 h of treatment (Figure 2). We found that CLytA-DAAO, in the presence of D-Alanine, is able to induce cell death in almost all cell lines tested, with a lower effect in the colon carcinoma cell line HT-29 (Figure 2A), pancreatic adenocarcinoma cell line Hs766T (Figure 2B), and the glioblastoma cell line HGUE-GB-42 (Figure 2C). 

**Figure 3:**
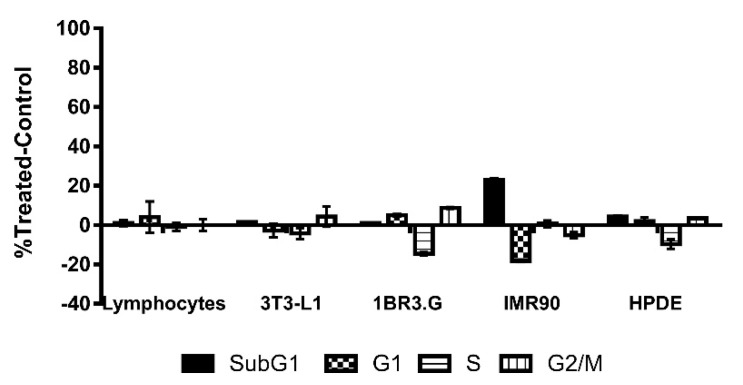
CLytA-DAAO effects on cell cycle distribution in non-tumor cell lines. Primary culture of lymphocytes, 3T3-L1 adipocytes, 1BR3.G, and IMR90 fibroblasts cell lines and HPDE human pancreatic cells were treated with 2 U mL^−1^ CLytA-DAAO and 1 mM D-Ala for 24 h and cell cycle distribution was determined by flow cytometry. Data represent the mean ± SEM of the percentages of treated cells minus those of the control untreated cells with *n* ≥ 3.

To determine the specificity of CLytA-DAAO treatment for tumoral cells, we determined CLytA-DAAO toxicity on non-tumor cell lines. We tested the CLytA-DAAO effect on lymphocytes primary cultures from non-oncological patients at HGUE, in mouse 3T3-L1 adipocytes, and in human non-tumoral cell lines from fibroblast (IMR90, 1BR3.G) and pancreas (HPDE). Results in Figure 3 show that CLytA-DAAO treatment only induced a minimal cell death in the IMR90 cells.

### 3.2. CLytA-DAAO Effect as Free and Bound to MNPs

**Figure 4:**
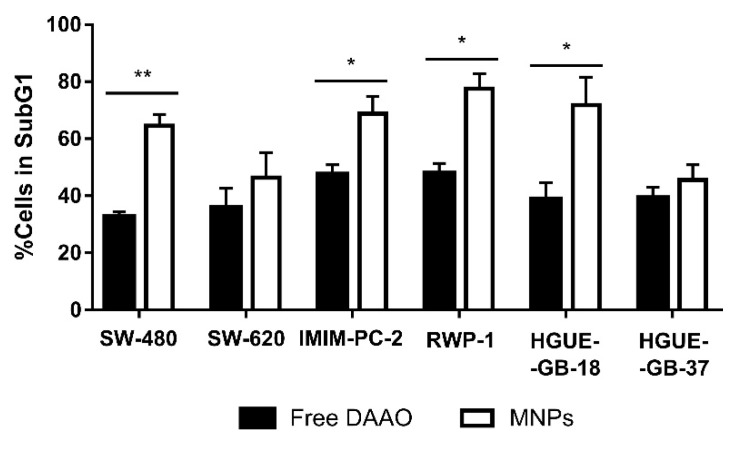
SW-480 and SW-620 colon carcinoma cell lines, IMIM-PC-2 and RWP-1 pancreatic adenocarcinoma cell lines, and HGUE-GB-18 and HGUE-GB-37 glioblastoma cell lines were treated with 2 U mL^−1^ free or bound to magnetic nanoparticles (MNPs) CLytA-DAAO, and 1 mM D-Ala for 24 h and cell cycle distribution was determined by flow cytometry. Data represent the mean ± SEM of the percentages of treated cells in SubG1 phase minus those of the control cells with *n* ≥ 3. * indicates *p* < 0.05 and ** *p* < 0.01.

In order to use an enzymatic therapy against cancer, it could be necessary to immobilize the enzyme in a support that prevents its degradation by the organism and allows us to direct the enzyme towards the tumor. As previously mentioned, MNPs have been used for immobilization of the CLytA-DAAO chimera. To determine whether immobilized CLytA-DAAO was as effective as free CLytA-DAAO, a cell cycle analysis was performed after treating cell lines for 24 h. Figure 4 shows the percentage of cells in the subG1 phase after receiving the treatment. Our results show that bound CLytA-DAAO was significantly more effective inducing cell death than CLytA-DAAO free enzyme.

### 3.3. Mechanism of Action of CLytA-DAAO

**Figure 5:**
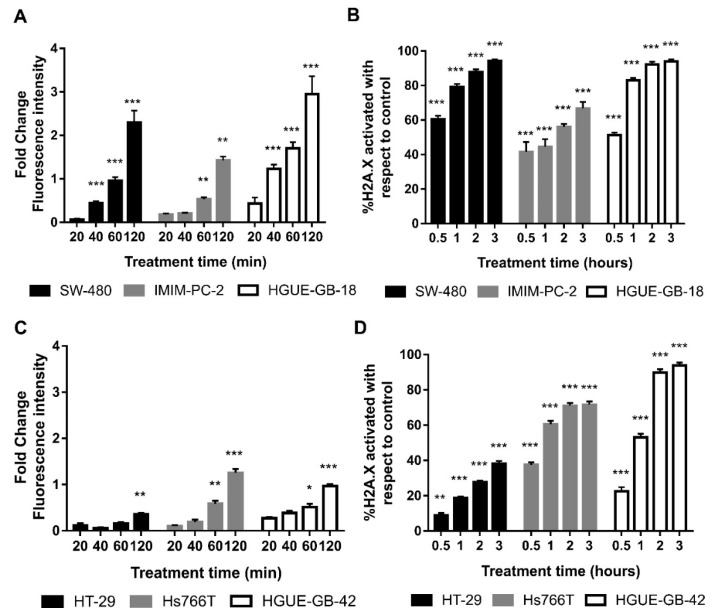
Oxidative damage induced by CLytA-DAAO. (**A**) SW-480 colon carcinoma cell line, IMIM-PC-2 pancreatic adenocarcinoma cell line, and HGUE-GB-18 glioblastoma cell line were treated with 2 U mL^−1^ CLytA-DAAO and 1 mM D-Ala for 20, 40, 60, and 120 min. Then, free radical production was determined using 2′,7′-dichlorodihydrofluorescein diacetate (H_2_DCF-DA). Values represent the fold change of fluorescence intensity compared to control; (**B**) SW-480 colon carcinoma cell line, IMIM-PC-2 pancreatic adenocarcinoma cell line, and HGUE-GB-18 glioblastoma cell line were treated with 2 U mL^−1^ CLytA-DAAO and 1 mM D-Ala for 0.5–3 h and then H2A.X phosphorylation was determined by flow cytometry. (**C**) HT-29 colon carcinoma cell line, Hs766T pancreatic adenocarcinoma cell line, and HGUE-GB-42 glioblastoma cell line were treated with 2 U mL^−1^ CLytA-DAAO and 1 mM D-Ala for 20, 40, 60, and 120 min. Then, free radical production was determined using H_2_DCF-DA. Values represent the fold change of fluorescence intensity compared to control; (**D**) HT-29 colon carcinoma cell line, Hs766T pancreatic adenocarcinoma cell line, and HGUE-GB-42 glioblastoma cell line were treated with 2 U mL^−1^ CLytA-DAAO and 1 mM D-Ala for 0.5–3 h and then H2A.X phosphorylation was determined by flow cytometry Data represent the mean ± SEM of the percentage of cells, with *n* ≥ 3. * indicates *p* < 0.05, ** *p* < 0.01, and *** *p* < 0.001.

In the chemical reaction catalyzed by the DAAO enzyme, H_2_O_2_ is produced, and this finally generates free radicals, able to induce DNA damage and cell death [37,38,39]. In order to test if this could be the mechanism involved in cell death, we determined the production of free radicals after CLytA-DAAO treatment in colon carcinoma, pancreatic adenocarcinoma, and glioblastoma cell lines. Results in Figure 5A show that, in response to the enzymatic treatment, a rapid and sustained increase in free radical production is observed in SW-480, IMIM-PC-2, and HGUE-GB-18. This result is extended to the rest of cell lines sensitive to cell death induced by CLytA-DAAO (data not shown). Next, we determined histone H2A.X activation in the same cell lines after the treatment with CLytA-DAAO, as this protein is phosphorylated in response to DNA damage. In Figure 5B, we observed an increase in histone H2A.X phosphorylation in all cell lines sensitive to cell death induction by CLytA-DAAO. Activation of histone H2A.X, clearly correlates with the increase in free radicals, thus the increase in ROS production is inducing DNA damage.

We also wanted to study whether the resistance to cell death induced by CLytA-DAAO observed in HT-29, Hs766T, and HGUE-GB-42 cell lines, could be explained by a lower generation of ROS or a lower DNA damage. In order to answer this question, ROS production (Figure 5C) and histone H2A.X phosphorylation (Figure 5D) were studied in these cell lines. The results observed do not allow a single conclusion of why these cell lines are resistant to the cell death induced by CLytA-DAAO. In the case of HT-29 colon carcinoma cell line, a lower production of free radicals and DNA damage can be observed in comparison with the sensitive cell lines. However, in Hs766T and HGUE-GB-42 cell lines, although free radicals do not increase as much as in sensitive cell lines, histone H2A.X phosphorylation reaches the levels observed in sensitive cell lines. 

### 3.4. Mechanism of Cell Death Induced by CLytA-DAAO

**Figure 6:**
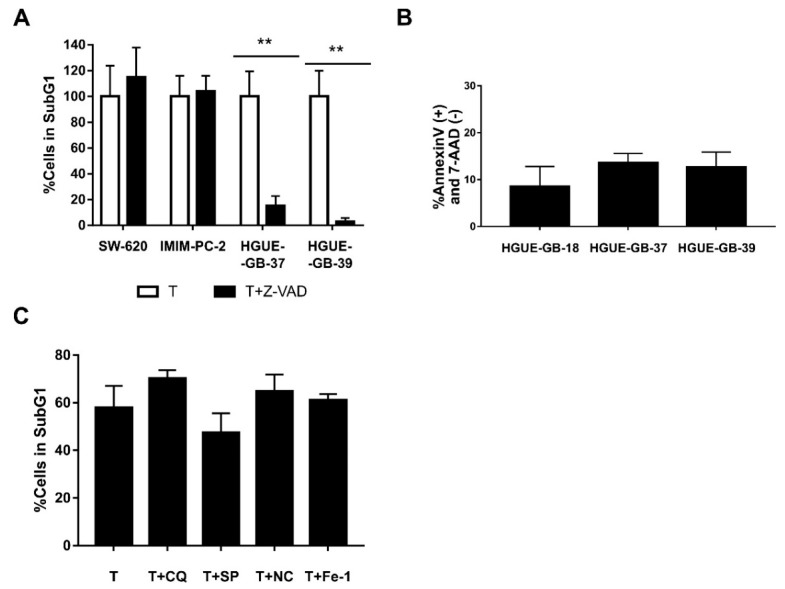
Type of cell death induced by CLytA-DAAO. (**A**) SW-620, IMIM-PC-2, HGUE-GB-37, and HGUE-GB-39 cell lines were treated with 2 U mL^−1^ free CLytA-DAAO, and 1 mM D-Ala in the presence or absence of 25 µM Z-VAD for 24 h and cell cycle distribution was determined by flow cytometry. (**B**) HGUE-GB-18, HGUE-GB-37, and HGUE-GB-39 glioblastoma cell lines were treated with 2 U mL^−1^ CLytA-DAAO, and 1 mM D-Ala, for 24 h and apoptotic cell death was determined through Annexin V-PE (+) and 7-AAD (−) labeling by flow cytometry. (**C**) IMIM-PC-2 cell line was treated with 2 U mL^−1^ CLytA-DAAO, and 1 mM D-Ala in the presence or absence of 10 µM chloroquine (CQ), 10 µM spautine-1 (SP), 20 µM necrostatine-1 (NC), and 10 µM ferrostatin-1 for 24 h and cell cycle distribution was determined by flow cytometry. Data represent the percentage of cells in sub-G1 phase, normalizing the treatment with CLytA-DAAO as 100% ± SEM with *n* ≥ 3. ** indicates *p* < 0.01.

In order to study whether CLytA-DAAO induced cell death was a caspase-dependent phenomenon, a pan-caspase inhibitor (Z-VAD) was used in combination with the treatment of CLytA-DAAO and D-Alanine. Figure 6A shows an example of this type of experiment in colon carcinoma (SW-620), pancreatic adenocarcinoma (IMIM-PC-2), and glioblastoma (HGUE-GB-37, HGUE-GB-39) cell lines. Our results indicate that only in glioblastoma, the Z-VAD was able to block cell death, this was also observed for all the other glioblastoma cellular models (data not shown). This result suggests that, in this type of tumor, CLytA-DAAO induces cell death through a caspase mediated apoptotic mechanism, while in colon and pancreatic cancer the cell death seems to be unrelated to the traditional apoptotic programmed cell death. To confirm the apoptotic cell death in glioblastoma, we carried out studies with Annexin V to determine whether phosphatidylserine externalization occurs. Figure 6B shows the percentage of cells treated with CLytA-DAAO and D-Alanine that were labeled with Annexin-PE but not with 7-AAD, that is, those that have externalized phosphatidylserine but whose membrane has not yet been affected. This result confirms that the cell death generated by CLytA-DAAO and D-Alanine in glioblastoma is apoptotic. 

To check whether Bax is playing a role in CLytA-DAAO induced cell death, we used CLytA-DAAO and D-Alanine in combination with a Bax-inhibiting peptide in colon, pancreatic, and glioblastoma cell lines, only a 25% decrease in cell death in the HGUE-GB-37 glioblastoma cell line was observed (Figure S1). This result demonstrates that the apoptotic and non-apoptotic cell death caused by CLytA-DAAO is mostly independent of Bax. 

Then, we evaluated whether other types of cell death inhibitors where able to inhibit CLytA-DAAO-induced cell death in colon and pancreatic carcinomas. Inhibitors against autophagic cell death, such as chloroquine or spautine-1, against necroptosis cell death, such as necrostatine-1, or against ferroptosis cell death, such as ferrostatine-1, were tested. In the Figure 6C, we show a representative result in the pancreatic adenocarcinoma cell line IMIM-PC-2, and we can observe that none of the inhibitors had a statistically significant effect on CLytA-DAAO-induced cell death. The same result was obtained in the rest of cell lines tested (data not shown). 

**Figure 7:**
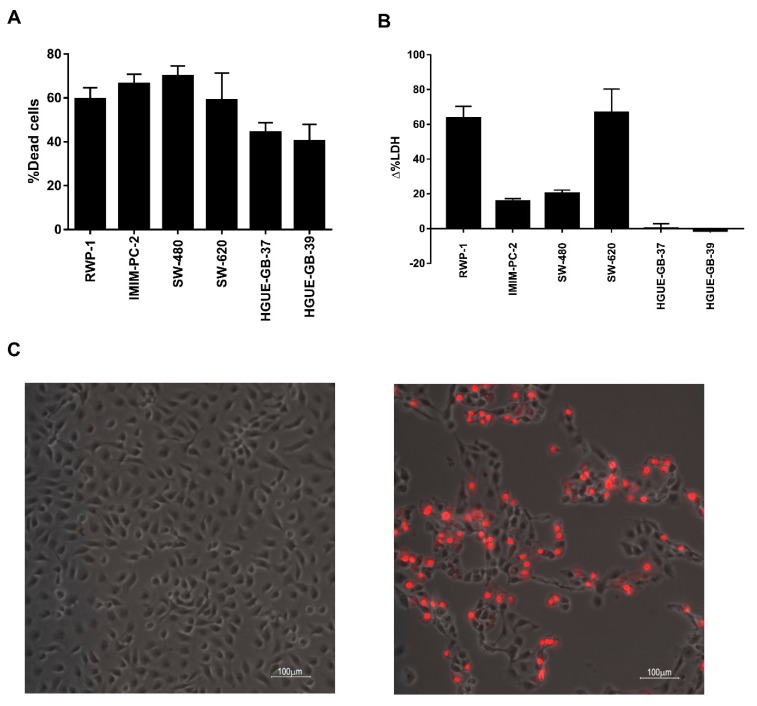
CLytA-DAAO plasmatic membrane rupture. (**A**) RWP-1 and IMIM-PC-2 pancreatic adenocarcinoma cell lines, SW-480 and SW-620 colon carcinoma cell lines, and HGUE-GB-37 and HGUE-GB-39 glioblastoma cell lines were treated with 2 U mL^−1^ CLytA-DAAO, and 1 mM D-Ala for 24 h and viability was determined by flow cytometry. Data represent the mean ± SEM of the percentage of dead cells with respect to control, with *n* ≥ 3. (**B**) RWP-1 and IMIM-PC-2 pancreatic adenocarcinoma cell lines, SW-480 and SW-620 colon carcinoma cell lines, and HGUE-GB-37 and HGUE-GB-39 glioblastoma cell lines were treated with 2 U mL^−1^ CLytA-DAAO, and 1 mM D-Ala for 24 h and extracellular LDH activity was measured. Data represent the mean ± SEM of the percentage of change in extracellular detected LDH activity between the treated cells and the control untreated cells, with *n* ≥ 3. (**C**) IMIM-PC-2 pancreatic adenocarcinoma cell line was treated with 2 U mL^−1^ CLytA-DAAO, and 1 mM D-Ala for 6 h, and propidium iodide uptake was determined by microscopy. The left panel shows control cells and the right panel shows treated cells.

Based on our previously shown results, we hypothesized that in pancreatic and colon carcinoma the death induced by CLytA-DAAO was a necrosis while in glioblastoma was a classic apoptosis. To test this hypothesis, we performed a released LDH activity test that is indicative of a necrotic cell death. In Figure 7, we compare CLytA-DAAO induced cell death (Figure 7A) with LDH extracellular activity (Figure 7B) in RWP-1 and IMIM-PC-2 pancreatic carcinoma cell lines, SW-620 and SW-480 colon carcinoma cell lines, and HGUE-GB-37 and HGUE-GB-39 glioblastoma cell lines. Our results show that in pancreatic and colon carcinoma cell lines, LDH extracellular activity is detected, suggesting a necrotic type of cell death. Meanwhile, no LDH extracellular activity was detected in the glioblastoma cell lines, suggesting again that the cell death induced by the enzyme in glioblastoma is a classical apoptosis.

Next, we tested the plasmatic membrane integrity using a different approach. In this case, we added to cells propidium iodide, after being treated with CLytA-DAAO for 6 h. In Figure 7C, we show a representative result in the IMIM-PC-2 cell line, which indicates that the plasmatic membrane rupture takes place. The same result was obtained in the rest of colon and pancreatic carcinoma cell lines tested but not in glioblastoma cell lines (data not shown).

### 3.5. CLytA-DAAO as an Enhancer of Other Treatments

A possible use of CLytA-DAAO as enzymatic therapy is its combination with classic treatments used in clinical practice, with the aim to enhance their effect. In this study, we combined a low dose of CLytA-DAAO with TSA, a histone deacetylase inhibitor, with radiotherapy and with PARP inhibitors, which are related to DNA repair [40]. 

**Figure 8:**
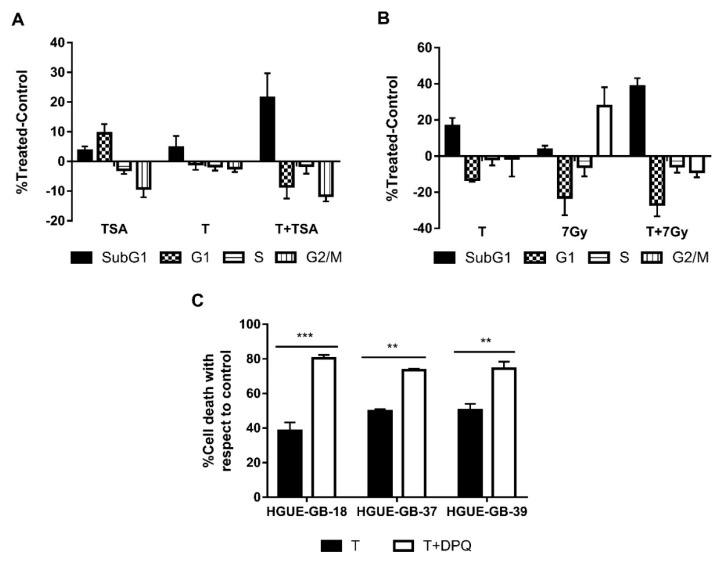
CLytA-DAAO as an enhancer of other treatments. (**A**) HGUE-GB-18 glioblastoma cell line was treated with 0.1 µM Trichostatin A (TSA) or 1 U mL^−1^ CLytA-DAAO and 1 mM D-Ala or a combination of both treatments over 24 h and cell cycle distribution was determined by flow cytometry. (**B**) RWP-1 pancreatic adenocarcinoma cell line was treated with 1 U mL^−1^ CLytA-DAAO and 1 mM D-Ala or 7 Gy radiotherapy or a combination of both treatments for 24 h and cell cycle distribution was determined by flow cytometry. (**C**) HGUE-GB-18, HGUE-GB-37, and HGUE-GB-39 glioblastoma cell lines were treated with 10 µM DPQ or 2 U mL^−1^ CLytA-DAAO and 1 mM D-Ala or a combination of both treatments over 24 h and viability was determined by flow cytometry. Data represent the mean ± SEM of the percentages of cells with respect to control, with *n* ≥ 3. ** indicates *p* < 0.01, and *** *p* < 0.001.

In order to verify whether we were able to potentiate the effect of TSA, we treated the cells with low doses of TSA and CLytA-DAAO either alone or in combination. In Figure 8A, we can observe the result obtained in the HGUE-GB-18 glioblastoma cell line. When we use low doses of TSA or CLytA-DAAO alone, we barely get 5% of cell death, however, when we combine both treatments, 20% of cell death is reached. This result was extended to the rest of the glioblastoma cell lines, although we did not observe potentiation in either pancreatic adenocarcinoma or colon carcinoma cell lines.

We performed the same experiment using radiotherapy at a 7 Gy dose, in Figure 8B we can observe that in the RWP-1 cell line, cell death increases combining both treatments. In the case of radiotherapy, we observed potentiation in all cell lines of exocrine pancreatic carcinoma and glioblastoma [41] (data not shown). 

Finally, as CLytA-DAAO generates DNA damage, we combined the treatment with CLytA-DAAO with DPQ, a PARP inhibitor. In Figure 8C, an enhancement of the CLytA-DAAO effect is observed when we combine the treatment with DPQ in glioblastoma cell lines. This result is not observed in the pancreatic or colon carcinoma cell lines (data not shown). 

## 4. Discussion 

In this article, we present data showing that an enzymatic therapy based on a CLytA-DAAO chimera constitutes a new therapeutic strategy for various types of tumors, so it can be used in colon carcinoma and, even more important, in pancreatic carcinoma and glioblastoma, two types of tumors for which there is no effective therapy to date.

Tumor cells are able to display several resistance mechanisms to the conventional therapies and, in many cases, the patients develop multiple resistance to drugs that are not related neither at the structural nor at the pharmacological level; this phenomenon is known as multidrug resistance (MDR) phenotype [42,43]. One strategy against chemotherapy resistance is the use of combination therapies with drugs not affected by the same resistance mechanism. The main problem of these combination therapies is how to administer them. In many cases, the different drugs cannot be administered simultaneously since the combination of their side effects could be very toxic for the patient [44,45]. 

It is generally accepted that if the anti-neoplasic drugs could be delivered and concentrated on the tumor, the side effects could decrease significantly [46,47]. Based on this, we can hypothesize that if the combination therapies are simultaneously delivered to the tumor, their side effects would decrease and, therefore, we would be able to use them at higher doses for systemic treatments. At this point, it could be even more interesting to concentrate inert compounds in the tumor and to turn them in situ in active drugs [4,5]. 

Initially, ROS-generating enzymes such as xanthine oxidase and glucose oxidase were used for such purposes [8]. However, the stability of these enzymes in vivo is low and their substrates (glucose, xanthine, and oxygen) are endogenous molecules whose concentration cannot be regulated. To overcome these limitations, the use of DAAO from *Rhodotorula gracilis* was proposed for cancer treatment [48]. 

On the other hand, another problem of cancer therapies is to ensure that the anti-neoplasic agents reach the tumor in an active form and at effective doses. The immobilization of DAAO in magnetic nanoparticles of Fe_3_O_4_ functionalized with 3-aminopropyltriethoxyxilane (APTES) was previously tested. However, this type of nanoparticle is made by glutaraldehyde following a relatively complex procedure and shows cytotoxicity [49]. In this work, we developed a delivery system for DAAO based on MNPs. One of the advantages of our method is that the enzyme-nanoparticles binding is not covalent, so the immobilization process is easy and straightforward. This was accomplished through generation of a chimeric protein: CLytA-DAAO in which the CLytA domain confers a high choline binding affinity [27]. Based on this, we immobilized the chimeric enzyme to MNPs functionalized with DEAE, a chemical compound structurally similar to choline. This binding allows an additional advantage, as the enzyme can be easily released from the nanoparticles using choline, once the enzyme reaches its therapeutic target. Another advantage is that the negative effects can be controlled by the addition of the enzyme substrate, D-amino acids, which are not found in the human proteins. For this reason, the nanoparticles harboring CLytA-DAAO are inert until the substrate was added. This could be made once the nanoparticles had been delivered to the tumor through a magnetic field. 

Our results show that CLytA-DAAO induces cell death in most of the cell lines tested (Figure 1 and Figure 2), and the enzyme bound to nanoparticles is more efficient as anti-neoplasic agent than the free enzyme (Figure 4). It is known that the optimal temperature of CLytA-DAAO is close to 25 °C [50], and one of the advantages of enzyme immobilization is the increase of their stability and their pH or temperature working-range [10,11]. Therefore, the reason why the enzyme is more efficient when it is immobilized is a higher biological stability of the enzyme at 37 °C (data not shown). 

We also wondered about the putative CLytA-DAAO toxicity on non-tumor cells. It is known that, due to its accelerated metabolism, cancer cells show increased levels of ROS in comparison with non-tumoral cells, so that cancer cells are more sensitive to death generated by oxidative stress [3]. The results in Figure 3 show that CLytA-DAAO did not significantly affect either peripheral blood lymphocytes primary cultures from non-oncological patients, mouse adipocytes, or human cell lines from fibroblast and pancreas. Therefore, our data suggest that CLytA-DAAO has either no effect on non-tumor cells or a significantly lower effect than in tumor cells. Although the underlying reasons for this lack of CLytA-DAAO effect on non-tumor cells remain unknown, it may be due to the fact that non-tumor cells are not in constant division and their hereditary material is less exposed to the effect of ROS [51] and also, unlike most tumor cell lines, have a TP53 wild type that protects them from the genomic instability generated by ROS [52]. The lack of effect in non-tumoral cells is really important since the low toxicity together with the additional advantages of CLytA-DAAO such as enzyme stereospecificity which allows to control enzymatic induction, and the non-covalent affinity binding of CLytA-DAAO to MNPs which allows the release in situ, confer to this enzymatic therapy interesting properties for clinical use.

Cell death induction correlates with the production of free radicals (Figure 5A) and the increase of DNA damage (Figure 5B) in all cell lines sensitive to cell death induced by CLytA-DAAO. Thus, DNA damage could be the final effector of CLytA-DAAO-induced cell death. In this figure, we also compare the increase of free radicals (Figure 5C) and DNA damage (Figure 5D) between the cell lines resistant to cell death induced by CLytA-DAAO. It is important to point out that we are talking about resistance to cell death induction, because in Table 1 it is obvious that Hs766T and HGUE-GB-42 are sensitive to the antiproliferative effect of CLytA-DAAO. However, as shown in Figure 2, minimal induction of cell death is observed in these cell lines, suggesting that the antiproliferative CLytA-DAAO effect in these cell lines is mostly cytostatic. HT-29 resistance could be related to a lower production of free radicals, but in Hs766T and HGUE-GB-42 cell lines there are an increase in ROS production and an activation of H2A.X histone. The differences observed between resistant cell lines suggest the presence of different CLytA-DAAO-resistance mechanisms. The putative mechanism present in HT-29 cells could be more focused on cell detoxification preventing ROS generation, for these reason, HT-29 is also resistant to the cytostatic effect of CLytA-DAAO as shown in Table 1. On the other hand, we also observed that the HT-29 cell line has the mutation V600E in BRAF [53], unlike the rest of the cell lines tested. This mutation has been associated with a poor prognostic and a greater resistance to the common therapies [54,55]. Therefore, this mutation could be the reason for the lower effect of CLytA-DAAO in this cell line. However, in Hs766T cells, the resistance mechanism could be based on the repair of DNA damage or the blockade of the mechanisms involved in DNA damage-induced cell death. It is interesting to mention that Hs766T cell line is quite resistant to several treatments, such as gemcitabine and others [56], and that this cell line has a wild type TP53 meanwhile IMIM-PC-2 and RWP-1 have mutant TP53. TP53 is crucial in the cellular response to agents that induce DNA damage [57,58]. In addition, it has been found that Hs766T cells have a mitotic defective checkpoint [59]. We know that initially CLytA-DAAO blocks the cell cycle in G2+M phase and then, these cells are committed to cell death (data not shown). Therefore, it could be that the incapacity of Hs766T cells to get blocked in G2+M is protecting them of the CLytA-DAAO induced cell death.

Results observed in Figure 5 raised the question of whether there is a universal molecular mechanism for CLytA-DAAO-induced cell death. To determine this, we treated different cell lines with CLytA-DAAO and D-Alanine either alone or combined with different cell death inhibitors, such as chloroquine or spautine-1 against autophagy, necrostatine-1 against necroptosis, ferrostatin-1 against ferroptosis, and a pan-caspase inhibitor, Z-VAD, against apoptosis. In colon and pancreas cancer cell lines, no inhibitor was able to block CLytA-DAAO-induced cell death (Figure 6A). Instead, we observed that Z-VAD is able to significantly block CLytA-DAAO-induced cell death in glioblastoma cell lines (Figure 6B). Consequently, in this type of tumor, CLytA-DAAO-induced cell death could be, at least in part, due to a classical apoptotic mechanism [60]. As a consequence, we thought that the cell death induced by CLytA-DAAO in colon and pancreas cell lines could be due to a necrotic mechanism. To verify this, we studied whether plasma membrane rupture occurred after treating the cells with CLytA-DAAO, and we observed that indeed, in the colon and pancreas carcinoma cell lines the plasma membrane rupture took place, but this did not occur in the glioblastoma cell lines (Figure 7). 

We also considered whether we could use the treatment with CLytA-DAAO as an enhancer of other treatments. TSA is an histone deacetylase (HDAC) inhibitor and therefore contributes to chromatin decompaction [61], thereby DNA would be more exposed to the effect of ROS and it is possible that the combination of low doses of TSA and CLytA-DAAO could have an enhanced effect. The result observed in Figure 8A confirmed our hypothesis in some cell models. On the other hand, one of the most commonly used therapies against cancer is radiotherapy. Radiotherapy generates DNA damage directly, due to ionizing radiation, and indirectly through the generation of ROS [62,63]. Since the cell death generated by CLytA-DAAO is due to the increase of ROS, we thought that we might be able to enhance the effect of radiotherapy using low doses of CLytA-DAAO. Results in Figure 8B show that it is possible to use CLytA-DAAO to enhance the radiotherapy effect. Since CLytA-DAAO causes the cell death through the generation of DNA damage, another option was to block DNA repair. To do this, we used DPQ, an inhibitor of PARP, in combination with CLytA-DAAO and D-Alanine. Again, we obtained a potentiation of cell death, although this result was only observed in glioblastoma cell lines (Figure 8C). It is important to point out that a recent report has shown that Olaparib, a PARP inhibitor, constitutes a useful therapy for a subset of pancreatic adenocarcinoma patients (8–9% of total patients), which show BRCA 1 and 2 alterations [64]. It is tempting to hypothesize that CLytA-DAAO could potentiate the Olaparib effect in such patients, however, neither IMIM-PC-2, RWP-1, nor Hs766T have been shown defective in BRCA 1 or 2.

Our results constitute a proof of concept suggesting that an enzymatic therapy based on MNPs-immobilized CLytA-DAAO could constitute a useful therapy in colon and pancreatic carcinoma as well as glioblastoma. Besides, it could be used as monotherapy and in combination with epigenetic and radiotherapy therapy and DNA repair base therapy. Besides, the advantages of these nanoparticles with respect to those previously used are that they do not show cytotoxicity, allowing tumor localization and low risk of side effects [65]. Then, antitumor activity could be triggered by the addition of a D-amino acid. Another advantage of our system is that the immobilization of the enzyme is not a covalent bond, so a controlled release of CLytA-DAAO from the nanoparticles is possible, once located on the tumor.

## 5. Conclusions

- A chimeric enzyme was built adding the CLytA domain to the D-amino acid oxidase (CLytA-DAAO) to allow its non-covalent binding to DEAE functionalized magnetic nanoparticles (MNPs).
- Free and immobilized CLytA-DAAO, in the presence of a D-amino acid, induce cell death in colon carcinoma, pancreatic adenocarcinoma, and glioblastoma cell lines.
- Non-tumoral cell models are not induced to death by the CLytA-DAAO chimera.
- CLytA-DAAO-induced cell death is caused by DNA damaged provoked by the increase of ROS.
- CLytA-DAAO-induced cell death in colon and pancreas carcinoma cell lines is a necrosis meanwhile that in glioblastoma cell lines is an apoptotic cell death.
- The therapy with CLytA-DAAO and D-Alanine can be used per se or as an enhancer of other treatments related to the generation of ROS or DNA damage, such as epigenetic inhibition of histone deacetylase, radiotherapy, and DNA repair inhibition.
- Our results show that treatment with CLytA-DAAO alone or in combination with other drugs may constitute a valid therapeutic alternative for poor prognosis tumors, such as exocrine pancreatic carcinomas and glioblastomas.
- We found three cell lines resistant to cell death induced by CLytA-DAAO: HT-29 colon carcinoma cell line, Hs766T pancreatic adenocarcinoma cell line, and HGUE-GB-42 glioblastoma cell line.

## Figures and Tables

**Table 1:** IC50 values (U mL^−1^) of free CLytA-D-amino acid oxidase (DAAO) with 1 mM D-Ala in different cell lines from colon carcinoma, pancreatic adenocarcinoma, and glioblastoma treated for 72 h.

Type of Tumor	Cell Line	IC50 CLytA-DAAO (U mL^−1^)
Colon carcinoma	SW-480	0.18 ± 0.06
	SW-620	0.34 ± 0.04
	HT-29	1.3 ± 0.54
Pancreatic adenocarcinoma	IMIM-PC-2	0.13 ± 0.02
	RWP-1	0.23 ± 0.06
	Hs766T	0.11 ± 0.03
Glioblastoma	HGUE-GB-18	0.38 ± 0.14
	HGUE-GB-37	0.28 ± 0.09
	HGUE-GB-42	0.36 ± 0.11

## Supplementary Materials

The following are available online at https://www.mdpi.com/2218-273X/10/2/222/s1, Figure S1: IMIM-PC-2, SW-480, HGUE-GB-18 and HGUE-GB-37 cells were treated with 2 U mL^−1^ free CLytA-DAAO and 1 mM D-Ala in the presence or absence of 200 μM of Bax-inhibiting peptide, V5 (BIP). Then, viability was determined by flow cytometry analysis. Data represents the percentage of CLytA-DAAO induced cell death in the presence of BIP taking the CLytA-DAAO induced cell death in the absence of BIP as the 100%. Data are presented as mean ± SEM with *n* ≥ 3. * indicates *p* < 0.05.
