# Supplementary material for: CLytA-DAAO, Free and Immobilized in Magnetic Nanoparticles, Induces Cell Death in Human Cancer Cells
Source: Biomolecules. 2020 Feb 3;10(2):222. doi: 10.3390/biom10020222 (PMC7072168; doi:10.3390/biom10020222)
Supplement: Supplementary file 1 [file biomolecules-10-00222-s001.pdf]

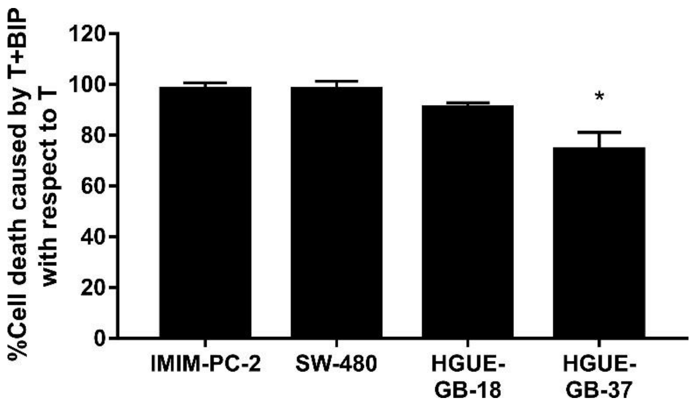

Figure S1. IMIM-PC-2, SW-480, HGUE-GB-18 and HGUE-GB-37 cells were treated with 2 U mL^{-1} free CLytA-DAAO and 1 mM D-Ala in the presence or absence of 200 μM of Bax-inhibiting peptide, V5 (BIP). Then, viability was determined by flow cytometry analysis. Data represents the percentage of CLytA-DAAO induced cell death in the presence of BIP taking the CLytA-DAAO induced cell death in the absence of BIP as the 100%. Data are presented as mean ± SEM with $n \geq 3$. * indicates $p < 0.05$.
